# GNOME: A Gaseous Nitrogen Oxide Measuring front-End for aqueous environmental materials

**DOI:** 10.1016/j.ohx.2023.e00459

**Published:** 2023-07-29

**Authors:** Samuel Bowman

**Affiliations:** Marine Chemistry and Geochemistry Department, Woods Hole Oceanographic Institution, 266 Woods Hole Rd., MS #25, Woods Hole, MA 02543, United States

**Keywords:** Nitrogen oxide, Nitrate, Nitrite, Vanadium sulfate reduction, Chemiluminescent analysis

## Abstract

GNOME is a straightforward and easy to build sample handling apparatus, designed to prepare dissolved nitrogen oxide species in aqueous environmental samples. GNOME is designed to serve as a sample preparation device for downstream chemiluminescent analysis. It is based on the familiar chemistry ring stand; the major advantage scalability designed to accommodate the needs of the user. Additionally, since GNOME is constructed of discrete, snap-to-fit components, the modular design allows users to easily substitute or replace parts. Given that there are few to zero commercial equivalents, construction plans to fill this hardware gap are offered herein. The inlet can resolve down to a lower limit of at least 0.05 nmoles NO_X_, and is instrumentally linear to a ≥10 nmoles NO_X_. The approach increases sample throughput, data quality, and overall user experience, making it more efficient than the off-the-shelf commercial equivalent.

## Graphical abstract

**Figure:**
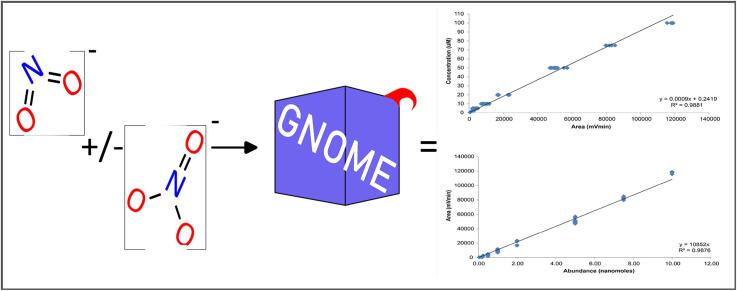

**Table:** 

Specifications table	
Hardware name	GNOME
Subject area	● Environmental, Planetary and Agricultural Sciences ● Chemistry and Biochemistry
Hardware type	● Chemical sample handling and preparation
Closest commercial analog	No commercial analog is available
Open Source License	GNU
Cost of Hardware	$1903.88
Source File Repository	Available with the article

## 1 Hardware in context

In many disciplines throughout the environmental sciences (e.g., biogeochemistry, chemical oceanography, freshwater hydrochemistry) determination of concentrations of dissolved ions in a water sample is centrally important. Among these analytes, dissolved nitrogen species are routinely measured to provide information about water quality, nutrient levels and sources of point source or non-point source pollution. Measurements of dissolved nitrogen oxides (NOx) species (namely nitrate (NO_3_^–^) and nitrite (NO_2_^–^)) routinely use several different approaches. Among the more common approaches in aqueous biogeochemistry laboratories, total NOx concentration or approximate species-specific concentrations are made by chemiluminescence after reduction to NO (e.g. [1], [2], [3], [4], [5]). This technique relies on reduction and liberation of the dissolved NO_X_ using acidic vanadium(III) [6], [7] to a gaseous NO phase. This is followed by introduction to a chemiluminescent analyzer where NO’s reaction with O_3_ generates photons that are quantified by photomultiplication [8] and recorded using a peak integrator [9]. The chemical reduction, gas liberation, liquid scrubbing, and flow control must be performed and monitored upstream of the chemiluminescent analyzer. It is therefore necessary to either build or buy a front end for conversion of nitrate/nitrite into NO prior to chemiluminescence measurement. Here, a design is introduced for this front end for chemiluminescent analyzers (GNOME). GNOME is a generalized inlet for chemiluminescent analyzers and does not preclude the downstream integration of any particular manufacturer. Here, build instructions are provided in hopes of addressing the limited number of commercially available equivalent NO_x_ sample preparatory devices. GNOME is licensed under the General Public License (GNU) stating that the ensuing information is free to sell/copy/modify so long as the resulting derivative product is licensed under the same conditions herein.

## 2 Hardware description

While the use of chemiluminescent analyzers for analysis of NOx by vanadium sulfate reduction is well established and there are many resources regarding reagents and specific models of instruments used, there is a relative paucity of available schematics regarding the gas handling front end for preparation of the NO_x_ itself. Additionally, ready-made commercial models are difficult to source and manufacturers of chemiluminescent analyzers may not provide or may have discontinued the production of an accompanying front end for their instruments (e.g. Teledyne API). Block diagrams [6], [8], [9], [10] are helpful in providing basic information on construction such a front end, but are limited in describing the actual parts needed, how the parts are interconnected, and how to modify or adjust the front end to maximize efficiency. One important consideration when selecting which instrument to use (commercial or homemade) is sample throughput-a parameter partially controlled by user experience and ergonomics. In conclusion, GNOME seeks to address both the lack of available gas handling schematics as well as improving upon sample throughput for the chemiluminescent analysis of aqueous environmental samples.

**Fig. 1:**
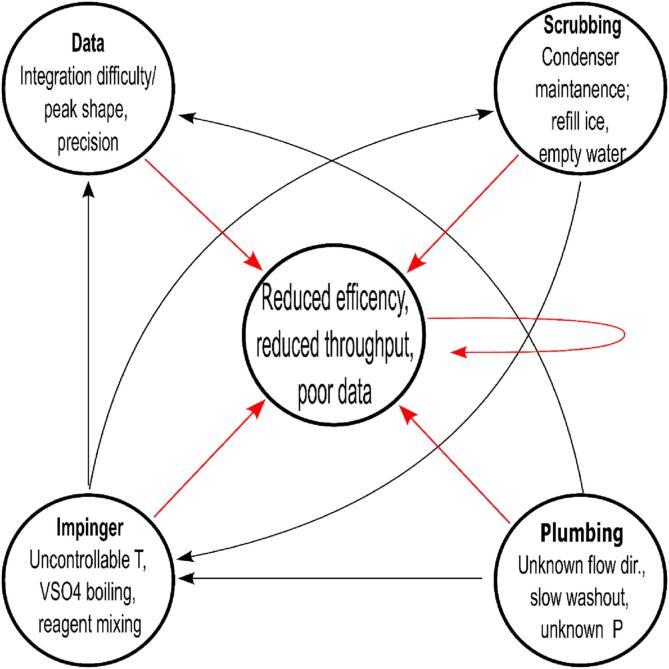
Empirical compounding issues resulting from a conventional NO_x_ sample handling apparatus. There are four groupings where issues arise and are organized as Data (output), Reaction cell chamber (reaction vessel containing acidic vanadium sulfate), Scrubbing (removal of liquid and acidic vapor from gas), and Plumbing (liquid and gas handling throughout system). Note the feedback loops occurring between various sections. These feedback loops further compound preexisting issues by multiplying them together, resulting in ever-increasing adverse effects. These effects manifest as efficiency loss, a reduction in sample throughput, and worse data quality. Poor data necessitates running replicates of samples, which, in turn continues and restarts the cycle.

The measurement of nitrate in particular may be hindered or complicated due to the constant 80 °C required for the NO release inside the reaction cell chamber [9]. Additionally, [9] mentions that the low throughput of 12 samples/hr may be doubled, but at the expense of precision, and fixed, microliter sample volumes (not acceptable for samples with a low NO_x_ concentration). To replace the heating element and thermocouple alone would cost in excess of $900 USD [11]. Fig. 1 illustrates additional compounding complications and disadvantages that are likely encountered using a conventional “block diagram” front end. GNOME improves upon conventional NOx Box front ends by addressing the issues listed in Table 1.

**Table 1:** Pitfalls encountered prior to the development of GNOME apparatus. P indicates and identified problem, and S indicates the corresponding solution. Note that the relationships and adverse positive feedback between the identified problems are presented pictorially in Fig. 1.

**Problem/Complication of Conventional Block Diagram Models**	**GNOME Resolution and Customization**
1P) Expensive heating unit/thermocouple is relatively difficult to replace if broken	1S) Less expensive heat tape saves money, improves temperature control, is easier to install and may be replaced
2P) Reaction cell chamber is contained inside an aluminum chamber hiding it from view	2S) Reaction cell chamber is exposed-allowing for visual inspection of reagent volume and possible reagent mixing
3P) Uncertain net flow direction can cause backflow of scrubbing reagents into reaction cell chamber-ruining vanadium sulfate and neutralizing acid	3S) 2-way positive/negative pressure gauge allows net flow to be adjusted with pinch-valve or needle valve
4P) Filter saturation could cause liquid to travel into chemiluminescent analyzer, causing significant damage	4S) Elevating filter above NaOH trap reduces the possibility of liquid into chemiluminescent analyzer
5P) Emptying and refilling ice bath reduces efficiency. If not emptied, water may overflow into NaOH scrubbing trap then proceed towards chemiluminescent analyzer. If ice is not replenished, condensation of vapor does not occur.	5S) Recirculating pump (e.g. chiller or cold water) or larger volume ice bath increases efficiency and user experience. Chiller ensures water temperature is suitable for condensation. Alternatively, for further cost-saving purposes, a 6 N NaOH ice bath may be used in-lieu of the recirculating pump. The chilled NaOH also serves to neutralize acidity.
6P) Conventionally described apparatus has a limited sample throughput.	6S) Sample throughput increased ∼ 184 % without sample peak overlap (Section 7.1).

GNOME differs from block diagram nitrogen oxide front ends in that it is a modular build, based around the familiar chemistry ring stand. The strengths of GNOME are chiefly its increase in ergonomics and efficiency; ultimately resulting in improved data quality (Fig. 1). By focusing on a modular design, the user may customize the respective components (reaction cell chamber, filter, chiller, etc.) that best fits their needs, and the modular framework itself may be scaled up or scaled down to fit the benchtop space available. Additionally, with the ease in which components can be removed from the system and replaced or swapped out for identical or similar parts makes GNOME more flexible than a prebuilt or rigidly designed unit with set-in components. By decreasing the time spent on maintaining the system, the user thereby increases their efficiency, their sample throughput, and thus, overall data quality. GNOME may be used for both conventional NOx analyses, as well as repurposed for additional-yet useful-laboratory tasks. Such uses and improvements include:

• greater flexibility and customizability due to the easily exchanged parts-allowing for multiple analyses to be performed beyond Σ[NO_x_]; e.g. [NO_3_^–^], [NO_2_^–^], and [I^-^] see [10];
• improved diagnostics and repair as the entire system does not need to be replaced if something stops working or sent to a third party for maintenance/technical support, thereby making GNOME more economical; and
• for projects requiring a vacuum line with condensers and filters, GNOME could be repurposed with minimal plumbing to be setup to perform additional tasks (e.g. sparging flasks, or solid–liquid separation via Büchner funnels).

## 3 Design Files

**Armature Schematic:** Figure for building the GNOME skeleton using 12.5 mm diameter aluminum rods-for parts in Bill of Materials a1-f10.

**Armature Attachments:** Figure illustrating the location of the GNOME attachments-for parts in Bill of Materials g1, g2, h1, h2, h3, h4, i1, i2, i3, j1, j2, k1, l1, l2, l3, l4, l5, m1, m2, m3, n1, n2, o1, and p1.

**Armature Components:** Figure illustrating the location of the GNOME components-for parts in Bill of Materials q1, r1, s1, t1, t2, u1, u2, u3, u4, u5, v1, v2, w1, w2, x1, x2, y1, y2, z1, z2, aa1, ab1, ac1, ad1, and ae1.

**Gas Handling 1-NaOH Trap-Filter:** Figure for building the gas handling line-for parts in Bill of Materials w1, af1, ag1, ah1, ai1, and aj1.

**Gas Handling 2-Condenser-NaOH Trap:** Figure for building the gas handling line-for parts in Bill of Materials w2, w3, and af2.

**Gas Handling 3-Reaction Cell Chamber-Condenser:** Figure for building the gas and vapor handling line-for parts in Bill of Materials w4, ad1, af3, aj2, and ak1.

**Gas Handling 4-Air Source Gauge-Reaction Cell Chamber:** Figure for building the gas handling line-for parts in Bill of Materials ac2, ac3, af4, and aj3, ak2, am1, an1, and at1.

**Gas Handling 5-Filter-Pressure Gauge:** Figure for building the gas handling line-for parts in Bill of Materials ac4, ac5, af5, ag2, ah2, ai2, aj4, an2, ao1, ap1, and aq1.

**Condenser Construction and Components:** Figure for building the condenser unit and inclusion of the optional recirculating chiller.

**Gas Handing 6-Carrier Gas Source-Air Source Gauge:** Figure for building the gas handling line-for parts in the Bill of Materials ar1, as1, ac6, and al1.

**All Gas Handling Lines Schematic:** Figure outlining the connection of the six gas handling lines to the major components.

**Plumbing Schematic:** Figure providing the overall layout for the GNOME apparatus as well as the optional recirculating chiller and building air/carrier gas source components.

**Completed GNOME:** Figure of the fully assembled GNOME apparatus with gas and liquid flow vectors.

## 4 Bill of materials

The Bill of Materials is included as Supplementary Information. The reaction cell chamber is a non-standard offering and was custom built; as such, the quoted price is an estimate. Many parts may be purchased from multiple online retailers and nearly identical parts may themselves be substituted for economic or availability reasons. The sources of the parts provide in the Bill of Materials is for new, unused parts (Table 2).

**Table 2:** The necessary Design Files for building GNOME.

Design file name	File type	Open source license	Location of the file
Fig. 2-Armature Schematic	Figure	GNU General Public License (GPL) 3.0	Available with the article
Fig. 3-Armature Attachments	Figure	GNU General Public License (GPL) 3.0	Available with the article
Fig. 4-Armature Components	Figure	GNU General Public License (GPL) 3.0	Available with the article
Fig. 5-Gas Handling 1-NaOH Trap-Filter	Figure	GNU General Public License (GPL) 3.0	Available with the article
Fig. 6-Gas Handling 2-Condenser-NaOH Trap	Figure	GNU General Public License (GPL) 3.0	Available with the article
Fig. 7-Gas Handling 3-Reaction Cell Chamber-Condenser	Figure	GNU General Public License (GPL) 3.0	Available with the article
Fig. 8-Gas Handling 4-Air Source Gauge-Reaction Cell Chamber	Figure	GNU General Public License (GPL) 3.0	Available with the article
Fig. 9-Gas Handling 5-Filter-Pressure Gauge	Figure	GNU General Public License (GPL) 3.0	Available with the article
Fig. 10-Condenser Construction and Components	Figure	GNU General Public License (GPL) 3.0	Available with the article
Fig. 11-Gas Handing 6-Carrier Gas Source-Air Source Gauge	Figure	GNU General Public License (GPL) 3.0	Available with the article
Fig. 12-All Gas Handling Lines Schematic	Figure	GNU General Public License (GPL) 3.0	Available with the article
Fig. 13-Plumbing Schematic	Figure	GNU General Public License (GPL) 3.0	Available with the article
Fig. 14-Completed GNOME	Figure	GNU General Public License (GPL) 3.0	Available with the article

## 5 Build instructions

**Fig. 2:**
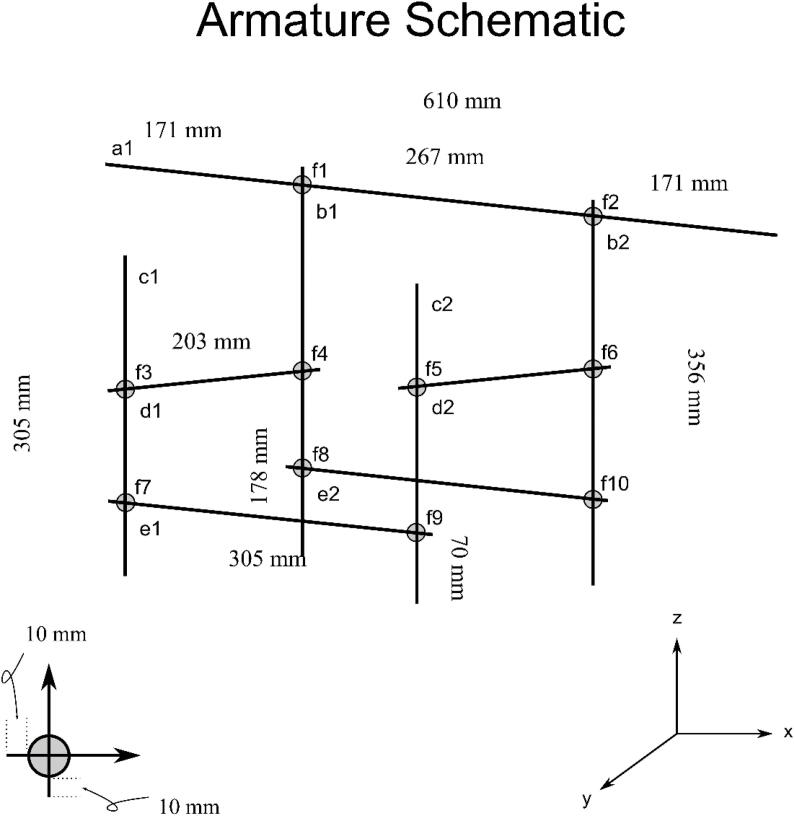
Armature Schematic. An illustration of the GNOME skeleton. Lowercase letter-number combinations correspond to parts a1-f10 in the Bill of Materials.

The instructions and accompanying figures are organized (not necessarily in numeric order) according to the general sample pathway from start to finish. It has been organized this way in hopes of making it clear that one section logically leads to the next. This is helpful when constructing the gas handling line (Fig. 2).

### 5.1 Armature skeleton

**Fig. 3:**
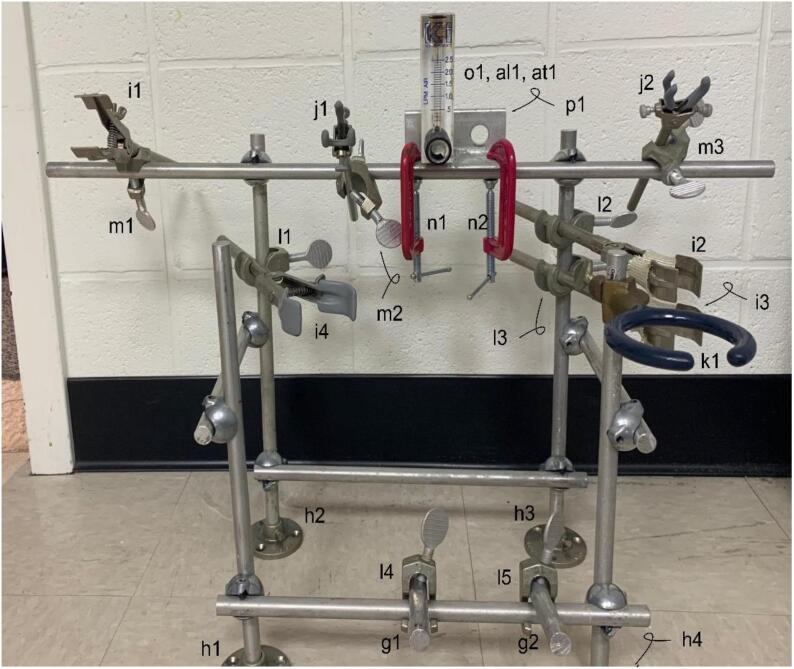
Armature Attachments. The ring stand skeleton that supports the components (Fig. 4); for parts g1, g2, h1, h2, h3, h4, i1, i2, i3, j1, j2, k1, l1, l2, l3, l4, l5, m1, m2, m3, n1, n2, o1, p1 in the Bill of Materials.

The 1/2{\Prime} aluminum rods (parts a1-e2) are easily cut to appropriate lengths as specified in Fig. 2 using a fine-tooth hacksaw blade or other suitable metal cutting tool. The lattice clamps (parts f1-f10) are then used to attach the 1/2{\Prime} aluminum rods together (Fig. 3).

### 5.2 Armature attachments

**Fig. 4:**
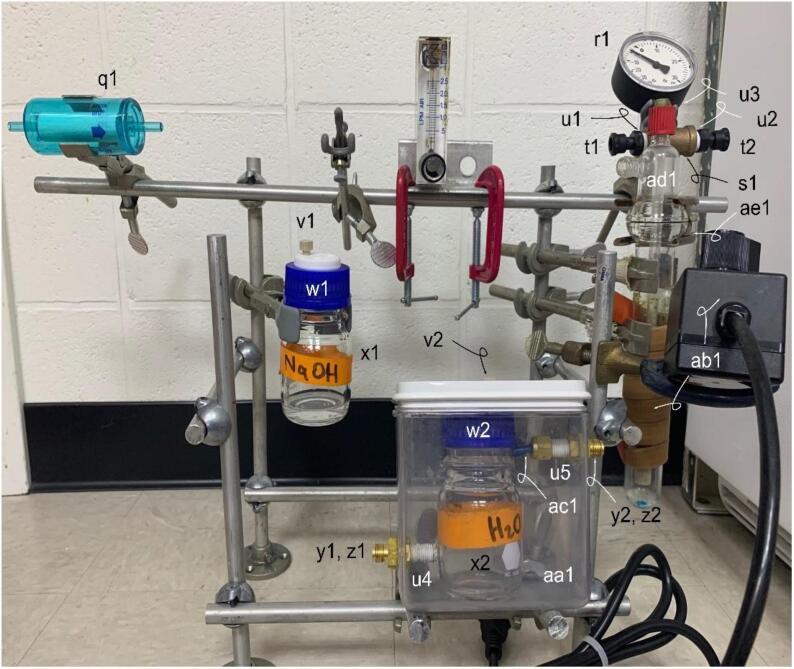
Armature Components. Gas and liquid handling components; for parts q1, r1, s1, t1, t2, u1, u2, u3, u4, u5, v1, v2, w1, w2, x1, x2, y1, y2, z1, z2, aa1, ab1, ac1, ad1, and ae1 in the Bill of Materials.

The armature attachments may be easily installed by following Fig. 3. However, it is worth noting that some of the attachments are positioned at specific locations on the armature skeleton. These include clamp holders m1, m2, and m3 are 9, 28, and 52 cm respectively from the left side of the aluminum rod. The hook clamp l1 is 10 cm from the top of the aluminum rod, while hook clamps l2 and l3 are 8 cm and 11 cm from the top of the aluminum rod respectively (Fig. 4).

A 1.39 cm (0.55{\Prime}) diameter hole is drilled in the steel plate p1. The bulkhead fitting al1 then allows for the air source gauge o1 to be mounted to the steel plate. Screw the bulkhead fitting al1 to the bottom port of the air gauge (gas source input) followed by the screwing the NPT side of fitting at1 into the top port of the air gauge (output). The output of o1 then connects to the reaction cell chamber (see Fig. 8). The front lower aluminum rod contains hook clamps l4 and l5 and are offset at 15 cm and 23 cm from the left of the aluminum rod and hold the ∼ 10 cm aluminum rods g1 and g2. This offset is to reduce the travel time (shorter tubing) of the incoming vapor from the reaction chamber to the condenser (Fig. 4, Fig. 10).

### 5.3 Armature components

#### 5.3.1 Major components

A dummy plug (v1 and v2) is used on the NaOH trap and the condenser collection bottle (see section 5.3.2) GL-45 caps to block off the unused port. The plug ensures there is no loss in sample gas, pressure drop, or outward spraying of hazardous hydroxide or hot vapor (from the condenser). The NaOH trap is located vertically below the filter to reduce the possibility of any liquid saturating the filter (hydraulic gradient) and entering the chemiluminescent analyzer downstream. The reaction cell chamber is a custom-built item. If such an exact replica cannot be built, the reaction cell is simply a three-ported glass vessel that is composed of two subunits. Each port is made for a GL-14 cap, but this type and diameter is not required. Additionally, a #25O-ring sits in the round bulb separating the top subunit from the bottom subunit. The top subunit contains two ports, one port is the carrier gas input and contains a fritted plunger, while the second port is the carrier gas + sample vapor output. The bottom subunit contains the remaining port and is used to introduce the liquid sample into the reaction cell chamber. The heating tape ab1 should be wrapped around the bottom half of the reaction vessel and the control box is allowed to rest on ring clamp k1 thereby protecting this electrical component from any incidental contact with liquids on the bench surface.

#### 5.3.2 Assembling the condenser (Fig. 10)

**Fig. 5:**
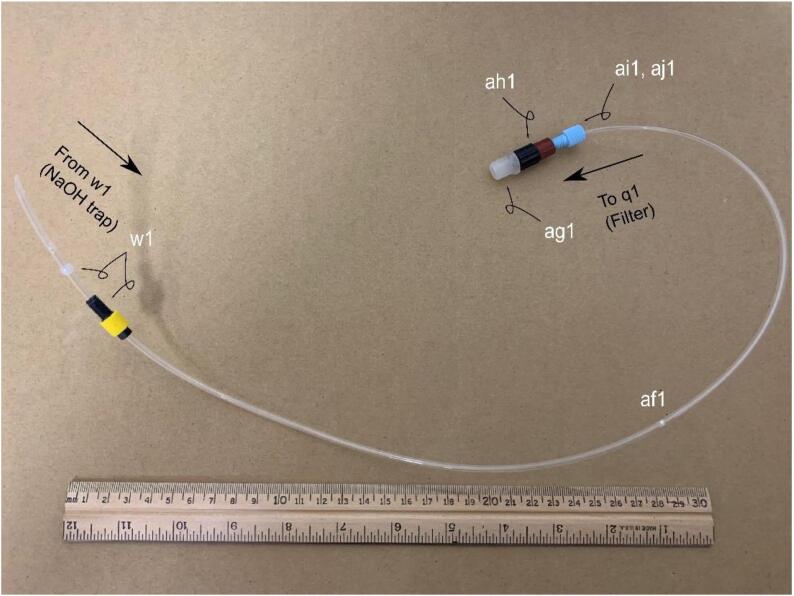
Gas Handling-1-NaOH Trap-Filter. Gas line plumbing for the NaOH scrubbing trap and the residual liquid/vapor filter; for parts w1, af1, ag1, ah1, ai1, and aj1 in the Bill of Materials.

**Fig. 6:**
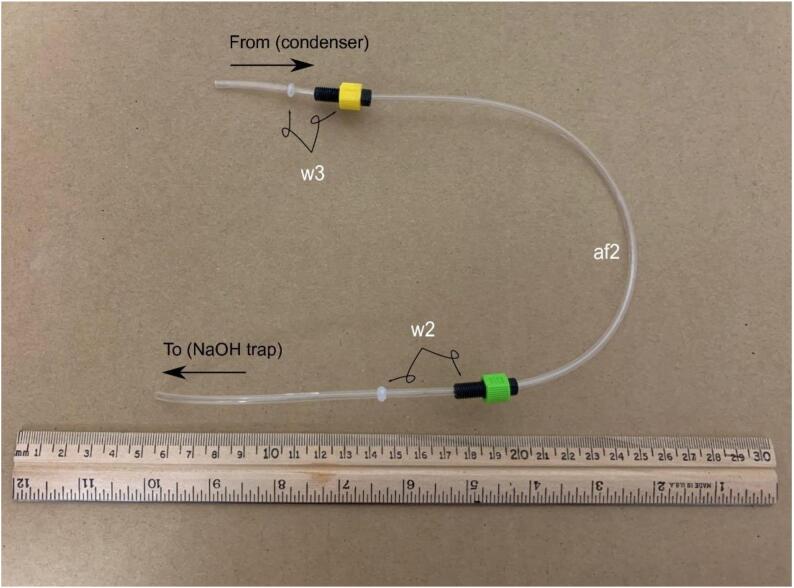
Gas Handling-2-Condenser-NaOH Trap. Gas line plumbing for the vapor condenser and NaOH scrubbing trap; for parts w2, w3, and af2 in the Bill of Materials.

**Fig. 7:**
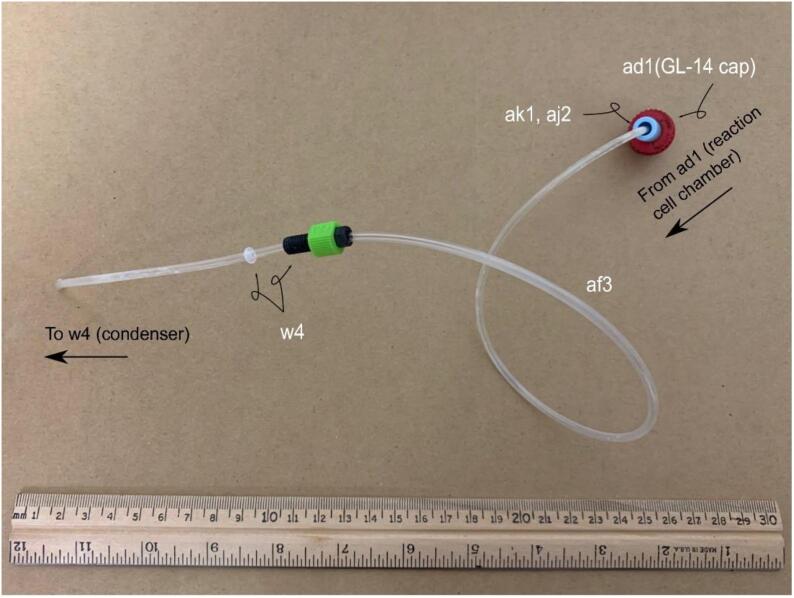
Gas Handling-3-Reaction Cell Chamber-Condenser. Gas line plumbing for the reaction cell chamber and the vapor condenser; for parts w4, ad1, af3, aj2, and ak1 in the Bill of Materials.

**Fig. 8:**
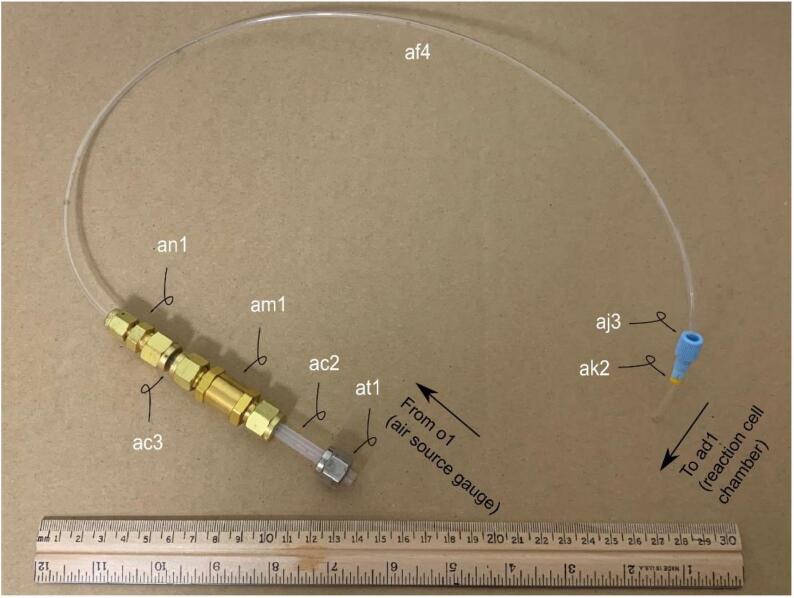
Gas Handling-4-Air Source Gauge-Reaction Cell Chamber. Gas line plumbing for the air source control gauge to the reaction chamber (reaction cell chamber); for parts ac2, ac3, af4, and aj3, ak2, am1, an1, and at1 in the Bill of Materials.

**Fig. 9:**
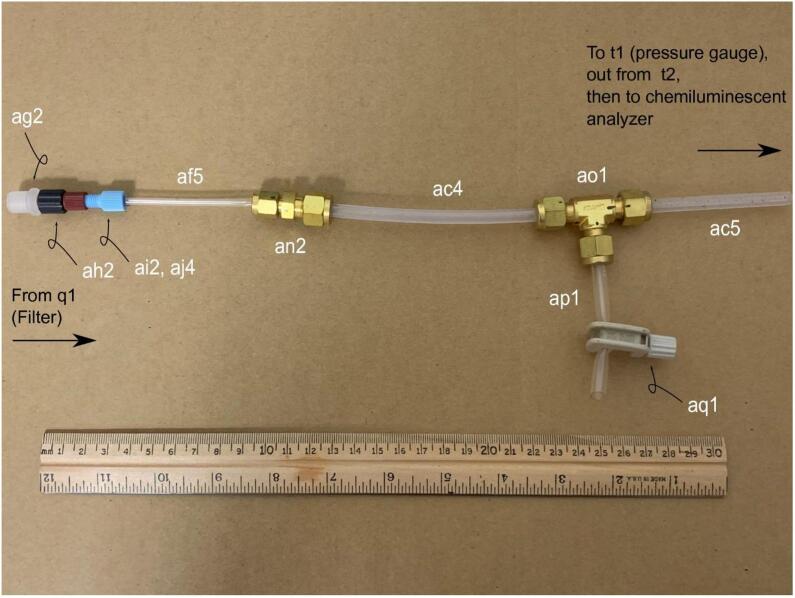
Gas Handling-5-Filter-Pressure Gauge. Gas line plumbing for the filter, pinch valve, and pressure gauge; for parts ac4, ac5, af5, ag2, ah2, ai2, aj4, an2, ao1, ap1, and aq1 in the Bill of Materials.

The suggested material of the condenser unit aa1 is a clear, easy to machine plastic. Opaque materials are not suggested as the condensate level would not be visible. Furthermore, since two 7/16{\Prime} diameter holes are drilled through the material, it is best to use a plastic which does not splinter or fracture which may lead to leaking. To reduce the likelihood of fracturing, start with a small pilot hole and gradually increase drill bit sizes until a 7/16{\Prime} diameter hole is reached. Apply a silicone sealant z1 and z2 to the bulkhead fittings y1 and y2. Insert a 3 cm long piece of 1/4{\Prime} nylon tubing to the incident cold water inlet y2. The purpose of this tubing is to reduce splashing. (Fig. 5, Fig. 6, Fig. 7, Fig. 8, Fig. 9, Fig. 11, Fig. 12).

**Fig. 10:**
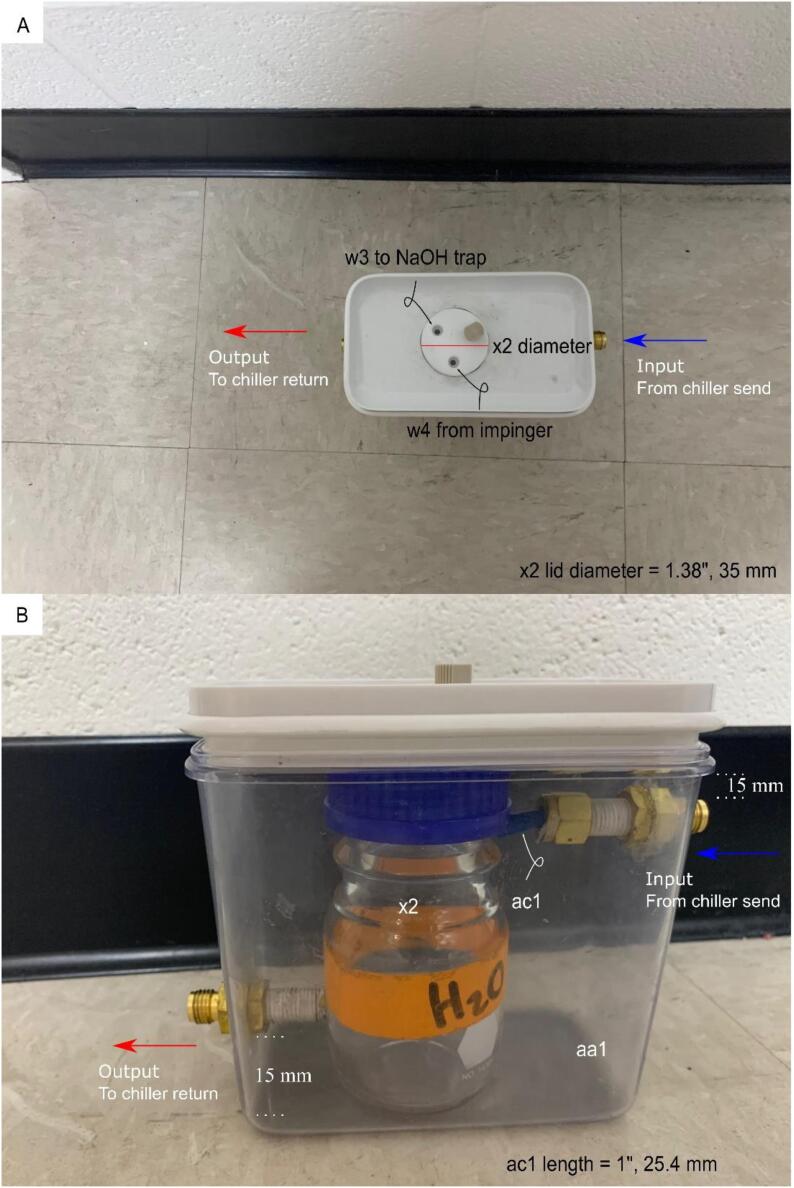
Condenser Construction and Components. A) Plan view of condenser. Note that there is a 1.38{\Prime} (35 mm) diameter hole drilled into the lid of part aa1. The lid of part x2 then becomes accessible for w4 from the upstream parts in Fig. 7. The gas + vapor output (from reaction cell chamber) in Fig. 6 connects to the gas + vapor input of x2 (lid of the condenser unit) using w4. The output from the condenser, w3, then carries the gas sans vapor to the NaOH trap. B) Part ac1 is used to prevent splashing, thus making the condensate (water) level in x2 easier to read. The incident, cold water from the recirculating chiller is stationed vertically above the outgoing, hot water. The hot water is sent back into the recirculating chiller, completing the circuit (Fig. 10). The apparent difference in positioning between the bulkhead fittings is the result of parallax, the incident port is 15 mm from the top of aa1, while the outgoing port is located 15 mm from the base of aa1.

**Fig. 11:**
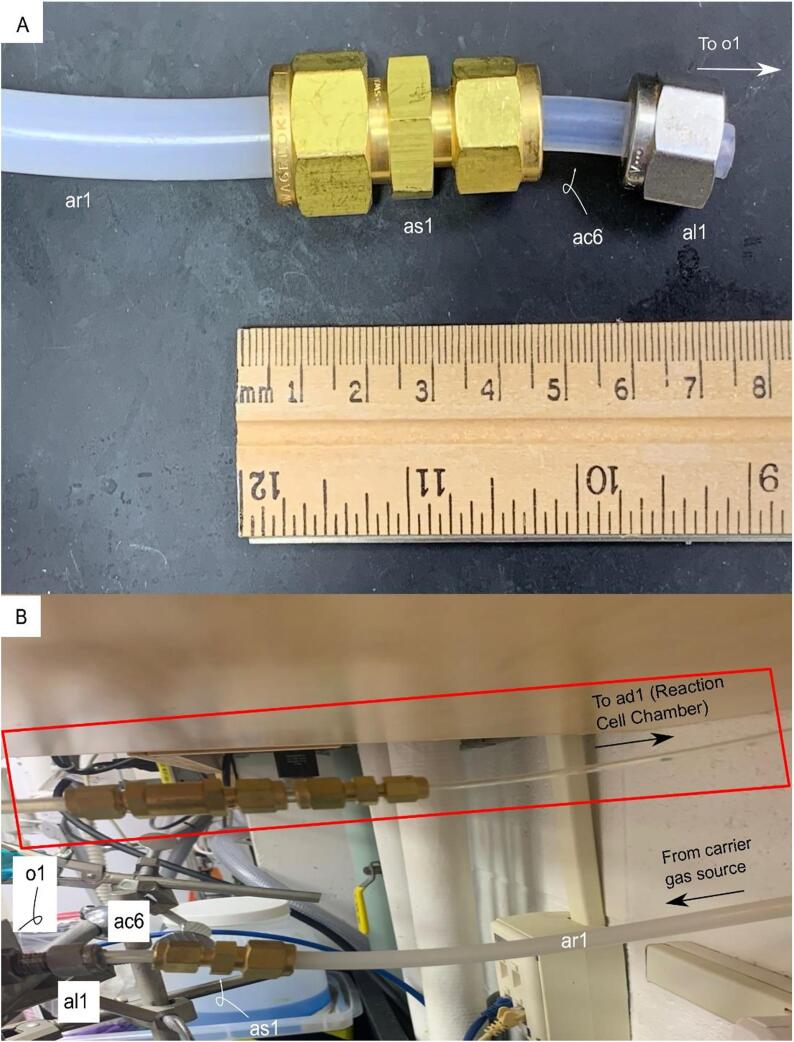
Gas Handing-6-Carrier Gas Source-Air Source Gauge. Gas plumbing line for the building air source to the air source control gauge. A) The incident building air or carrier gas supply passes through ar1 and is controlled by the air source gauge, o1 (Fig. 8). B) Photograph of the incident air source gas plumbing line, ar1, as1, ac6, and al1 in relation to the downstream air source gauge, o1. The red box indicates the gas plumbing line in Fig. 8.

**Fig. 12:**
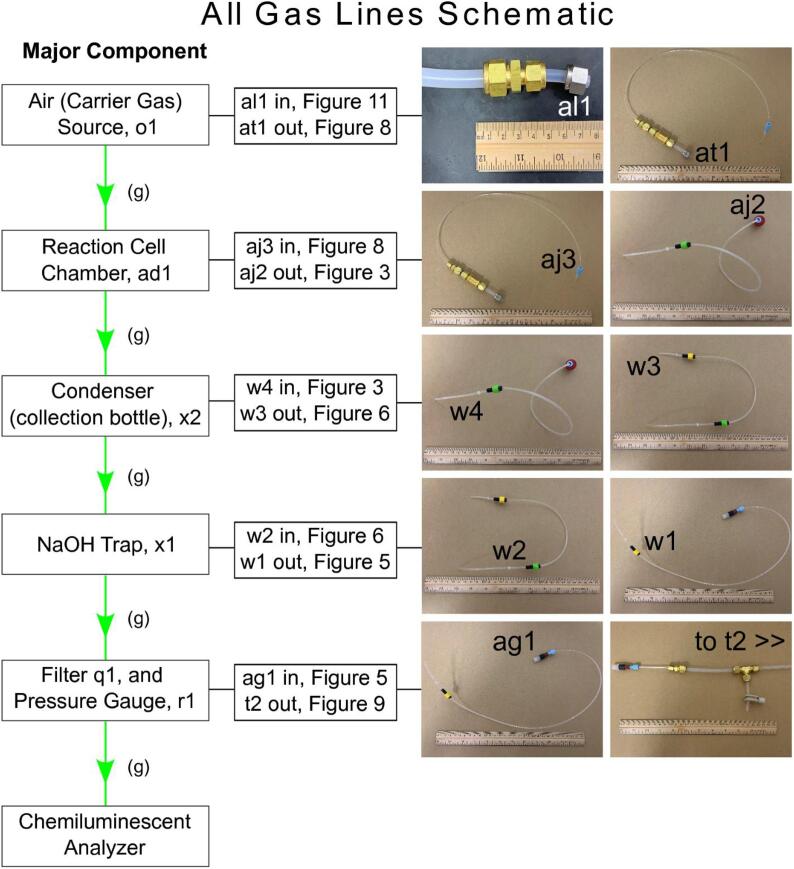
All Gas Handling Lines Schematic. An illustration of the six gas handling lines in Section 5.4. The green arrows in the left column indicate directional gas flow. The middle column presents the terminal parts (see Bill of Materials) of each gas handling line and are listed as either “in” or “out” and correspond to the input and output of the major components. The right column contains images of each gas handling line with the corresponding “in” or “out” terminal parts that relate to the Major Component.

### 5.4 Gas handling tubing

There are six segments of the gas handling tubing for GNOME. Lengths may be adjusted to fit the needs of the user, although it is advisable to keep the lengths of tubing as short as possible to reduce residence times and improve throughput. The assembly directions for each gas handling segment are not necessarily in the order of gas flow throughout GNOME but are instead ordered such that the parts of each segment broadly follows the Bill of Materials sequence.

#### 5.4.1 Assembly of the NaOH Trap-Filter (Fig. 5)

The NaOH trap-filter line af1 is a 60 cm piece of 1/8{\Prime} nylon tubing and connects the output of the NaOH trap to the input of the filter. It is important to ensure that the nylon tubing is above the NaOH in the trap to prevent sweeping any of the solution towards the filter, and ultimately towards the chemiluminescent detector. Cutting the end of the nylon tubing at an acute angle (Fig. 5) makes inserting through the 1/8{\Prime} opening of the VICI fitting and NaOH trap GL-45 cap w1 easier. (Fig. 6).

#### 5.4.2 Assembly of the condenser – NaOH trap line

The condenser-NaOH trap line af2 is a 40 cm piece of 1/8{\Prime} nylon tubing and connects the output of the condenser to the input of the NaOH trap. The end of the tubing that attaches to the NaOH trap (w2 side) should be submerged below the hydroxide to most effectively scrub the vapor. Conversely, the condenser side (w3) of the nylon tubing should be kept out of the condenser liquid to prevent dilution of the NaOH by condensate and to reduce the likelihood of liquid water or vapor travelling towards the chemiluminescent analyzer. (Fig. 7).

#### 5.4.3 Assembly of the reaction cell chamber – Condenser line

The reaction cell chamber-condenser line af3 is a 50 cm piece of 1/8{\Prime}nylon tubing that connects the output of the reaction cell chamber to the input of the condenser. The condenser side (w4 end) of the nylon tubing should extend to the base of the condenser bottle x2, aiding in condensation and reducing the likelihood of hot water vapor travelling towards the NaOH trap and towards the chemiluminescent analyzer. (Fig. 8).

#### 5.4.4 Assembly of the air source – Reaction cell chamber line

The air source-reaction cell chamber line af4 is a 65 cm piece of 1/8“ nylon tubing that connects the output of the air source gauge (via check valve am1 and reducing union an1) to the input of the reaction cell chamber (inserted 2 cm into reaction cell chamber). The air source side of the line utilizes a 1/8”-1/4“ reducing union to join the 65 cm 1/8” nylon tubing to a short (3 cm) piece of 1/4“ nylon tubing ac3. Connect the check valve to the air source gauge output o1 (with NPT-Swagelok 1/4” converter at1) using a 5 cm piece of 1/4” nylon tubing ac2. (Fig. 9).

#### 5.4.5 Assembly of the filter – Pressure gauge line

The filter-pressure gauge line connects the filter output to the pressure gauge component input and incorporates a bleed valve. A 10 cm piece of 1/8{\Prime} nylon tubing af5 connects to an 11 cm piece of 1/4{\Prime} piece of nylon tubing ac4 using a reducing union an2. The length of the 1/4{\Prime} Tygon tubing ap1 is unimportant, however the 1/4{\Prime} nylon tubing ac5 is 6 cm in length. Tygon is recommended because it is soft and can be easily compressed by the bleed pinch valve aq1. The downstream end of ac5 connects to the push–pull fitting t1 of the pressure gauge component. (Fig. 11).

#### 5.4.6 Assembly of the air source – Air gauge line

The air source-air gauge line ar1 connects the incident carrier gas (O_2_, N_2_, or compressed air) to the input of the air source gauge o1. The carrier gas line is a 3/8{\Prime} piece of nylon tubing and connects to the 1/4{\Prime} nylon tubing ac6 (as short as possible) via a reducing union as1. The length of the 3/8{\Prime} nylon tubing is determined on an ad hoc basis (distance from GNOME to carrier gas source). (Fig. 12).

#### 5.4.7 Final assembly of all gas handling line segments

**Fig. 13:**
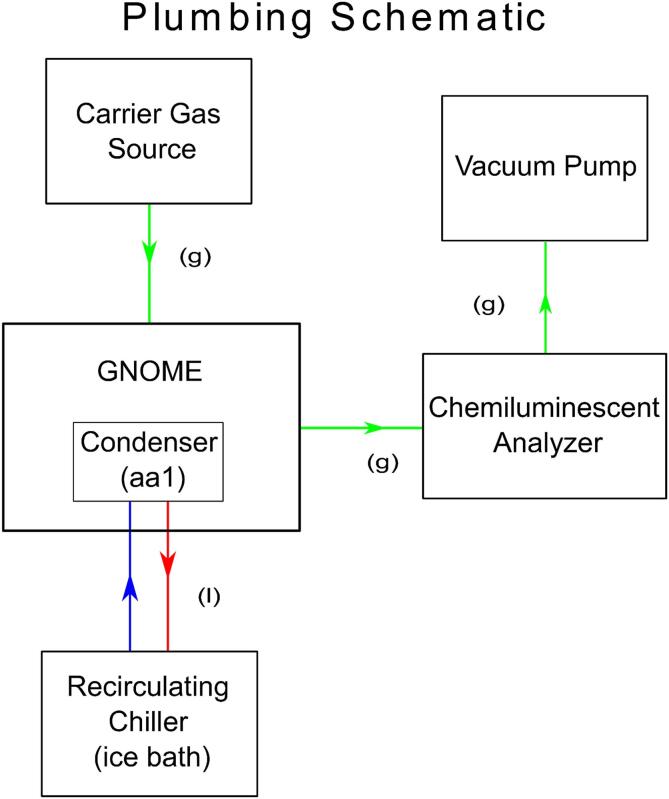
Plumbing Schematic. An illustration of the gas and liquid plumbing of GNOME and exogenous components. Green arrows indicate gas and vapor flow, blue and red arrows indicate cold (send) and hot (return) liquid flow with respect to the recirculating chiller.

Each of the six gas handling lines are connected to their respective component. The flow direction of the carrier gas is as follows; carrier gas source, air source gauge, reaction cell chamber, condenser collection bottle, NaOH trap, filter, pressure gauge. Following the pressure gauge, the dry sample + carrier gas enters the chemiluminescent analyzer. (Fig. 13).

### 5.5 Liquid handling condenser line

**Fig. 14:**
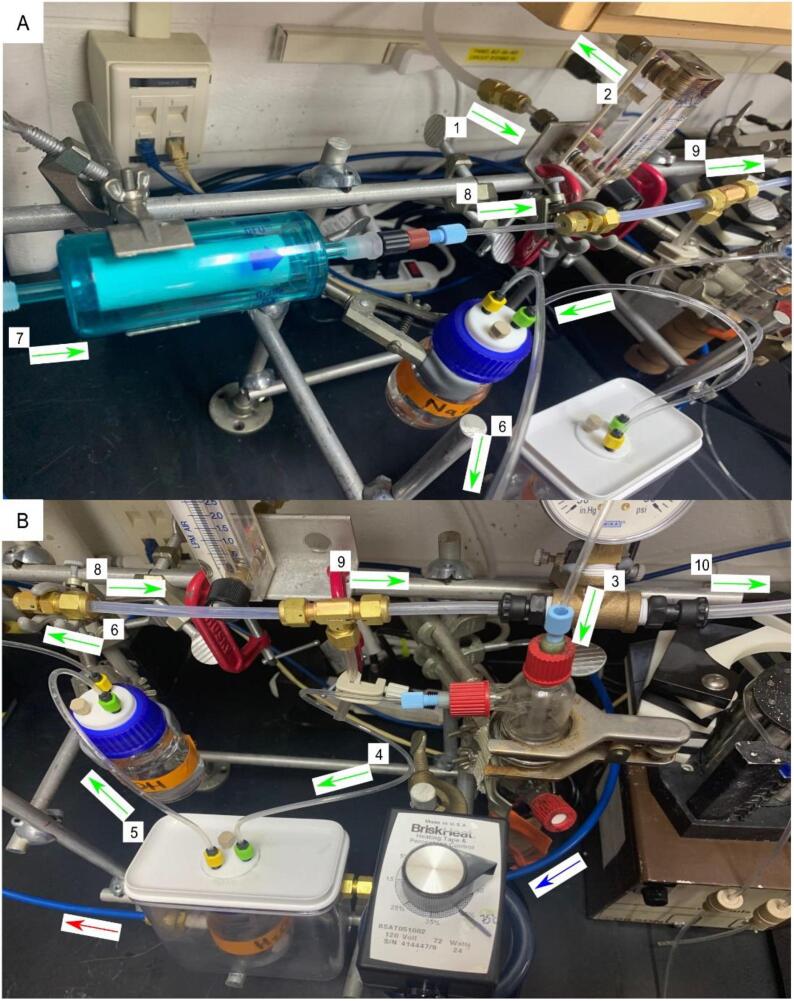
Completed GNOME. The green arrows correspond to the gas flow direction throughout GNOME. The numbers 1–10 associated with each of the green arrows correspond to the sequential flow direction (No.1 is most upstream-proximal to the carrier gas source, No.10 is most downstream-proximal to the chemiluminescent analyzer). In this configuration, the carrier gas source is building air (barb nozzle located ∼60 cm upstream of No.1), although a regulated gas cylinder may also be used. The red and blue arrows correspond to the respective hot and cold liquid flow directions of the condenser and recirculating chiller-located below GNOME under the standard-height benchtop. The vacuum pump is also adjacent to the recirculating chiller, with the pump inlet away from any condensate. Note that the vacuum pump is downstream of the chemiluminescent analyzer in the pathway, building air(No.1) → GNOME → chemiluminescent analyzer(No.10) → vacuum pump. The chemiluminescent analyzer is located only ∼50 cm offscreen to the right of No.10. A) Side view of GNOME. B) Alternate view: note the peristaltic pump located at the bottom right of the pane.

A chilling unit is required to condense the vapors from the reaction cell chamber. Examples include a simple ice bath or a recirculating chiller. Either of these examples is sufficient provided that the temperature is cold enough (∼5°C) to condense the vapors entering the condenser collection bottle x2. In this paper, we have opted to use a recirculating chiller. The condenser unit aa1 in Section 5.3.2 is designed for the attachment of a recirculating chiller (or recirculating ice bath if an external pump is installed). For users wishing to install a recirculating chiller, attach nylon tubing to both the recirculating chiller output/send and input/return. The lengths of the nylon tubing are determined on an ad hoc basis according to the needs of the user. However, to limit temperature interaction with the ambient air, the shortest length possible is recommended. If long lengths are required or to limit warming from the ambient air, foam tubing may be wrapped around the 1/4{\Prime} nylon tubing. The output/send from the recirculating chiller attaches to the bulkhead fitting y2 on the condenser unit aa1. The input/return to the recirculating chiller attaches to the bulkhead fitting y1 on the condenser unit aa1. (Fig. 14).

### 5.6 Completed GNOME

The completed GNOME is comprised of the skeleton armature, the hardware attachments, the major components, the gas handling lines, and liquid handling line (recirculating chiller). If the user wishes to further improve ergonomics, they may choose to use a peristaltic pump to send and receive the vanadyl sulfate to and from the reaction cell chamber via the GL-14 injection port. Alternatively, if the GL-14 cap is removed, the vanadyl sulfate may be extracted with a syringe and flexible (e.g. silicone) tubing attached. These approaches eliminate the need to unwrap the heating tape from the reaction cell chamber, and the physical removal of the reaction cell chamber from GNOME itself.

## 6 Operation instructions

Operation of GNOME is and outlined in a stepwise procedure below for both 6.1, 6.2. The procedure below is specified for the analysis of a “pure” sodium nitrate standard (although nitrite (if present) will also be reduced, so the resulting NO_x_ gas is a combination of nitrate and nitrite). It is important to reiterate, however, that environmental samples may not contain a single nitrogen oxide species, meaning that the measured NO_x_ gas is a multicomponent mixture.

### 6.1 Preparation

1) Turn on or open the carrier gas source (we recommend nitrogen (N_2_) as it will prolong the reductive capability of the vanadium), vacuum pump, recirculating chiller, and chemiluminescent analyzer (Fig. 13)
2) Adjust the pressure gauge r1 such that the air source positive pressure matches the negative pressure of the vacuum-the gauge is neutral
3) For a 500 mL stock of 2 % wt/v (0.122 N) acidic vanadyl sulfate solution; dissolve 9.95 g of vanadium sulfate in a 500 mL bottle of 2 N HCl
  a. Note: The oxidized vanadyl(IV) sulfate solution is bright blue in color
  b. Note: The toxicity associated with vanadium (III) may be avoided by use of alternate reducing reagents include Ti(III) or Fe(II) + Mo(IV) (see [12] or [9] respectively for more information).
4) Pour ∼ 80 mL of the acidic vanadyl(IV) sulfate solution into a flask and reduce the vanadium (IV) to vanadium(III) by mixing with elemental mossy zinc
  a. Note: The vanadium has been sufficiently reduced when the color of the solution is greyish-purple
  b. Note: It is possible to over reduce the vanadium solution. If the vanadium solution turns pink-purple in color, the redox state of the vanadium has become overly reduced and is now vanadium(II) [6].
  c. Selectivity of nitrite reduction to NO alone be accomplished by the employment of a 0.2 M sodium iodide and glacial acetic acid mixture [8], [13]. This method is exothermic and does not require a heating element [9]. It is worth noting, however, that a controllable heating element provides advantages of its own as well (Fig. 1).
5) Add ∼80 mL of the acidic vanadium(III) solution to the reaction cell chamber ad1
  a. Note: To avoid unwrapping the heat tape (Fig. 14) or disassembly of the reaction cell chamber, the solution may be introduced using either an external peristaltic pump or a syringe via the GL-14 injection port.
6) Add ∼60 mL of 1 N NaOH to the NaOH trap x1
7) To prepare the 10 mM sodium nitrate (NaNO_3_) standard solution, dissolve 0.084995 g of NaNO_3_ in 100 mL of NO_x_ free water. Dilute this stock solution to various size standards (e.g. 0.5 µM, 10 µM, 20 µM).

### 6.2 Analysis

1) Monitor the pressure gauge r1 throughout the entire NO_X_ analysis. Aim for a gas throughput rate of >450 mL/min, adjusting the air source gauge o1 and the pinch valve aq1 as needed to ensure a high throughput and pressure-balanced system.
2) Wait until the heat tape ab1 has raised the temperature of the acidic vanadium solution to the desired temperature (e.g. 65–80C) and for the water in the condenser aa1 to be cooled to ∼5C.
3) Ensure that the background [NO_X_] has stabilized prior to standard or sample injection.
  a. Note: If GNOME is opened or exposed to the laboratory air the [NO_X_] will be temporarily affected, wait for the system to settle before continuing with injections.
4) Inject a known volume (e.g. 100 µL) of a sodium nitrate standard solution (e.g. 100 µM) into the reaction cell chamber ad1 using the GL-14 injection port with inserted septa.
5) Wait for the injected standard to enter the chemiluminescent analyzer and integration software (∼15–17 s). The travel time is dependent upon the total length of all tubing in GNOME (some users may experience slower or faster washout times depending on the physical distance between their GNOME and chemiluminescent analyzer).
6) Repeat Steps 7–8 for a range of sizes of sodium nitrate standards (0.5 µM, 10 µM, 20 µM). Proceed with sample injections once a suitable calibration curve of the standards is complete.

### 6.3 Shutdown

1) Turn off the recirculating chiller and the chemiluminescent analyzer
2) Turn off the vacuum pump
3) Ensure that the pressure gauge r1 reads a positive pressure
  a. Note: This is important as a negative pressure can pull NaOH into the condenser collection bottle x2, and then eventually into the reaction cell chamber ad1-ruining the vanadyl sulfate reagent.
4) Vent GNOME to equalize it with the ambient laboratory air pressure
  a. Note: To vent, simply loosen one of the GL-14 caps on the reaction cell chamber as well as the GL-45 cap on the NaOH trap x1.
5) Close the carrier gas source

## 7 Validation and characterization

**Fig. 15:**
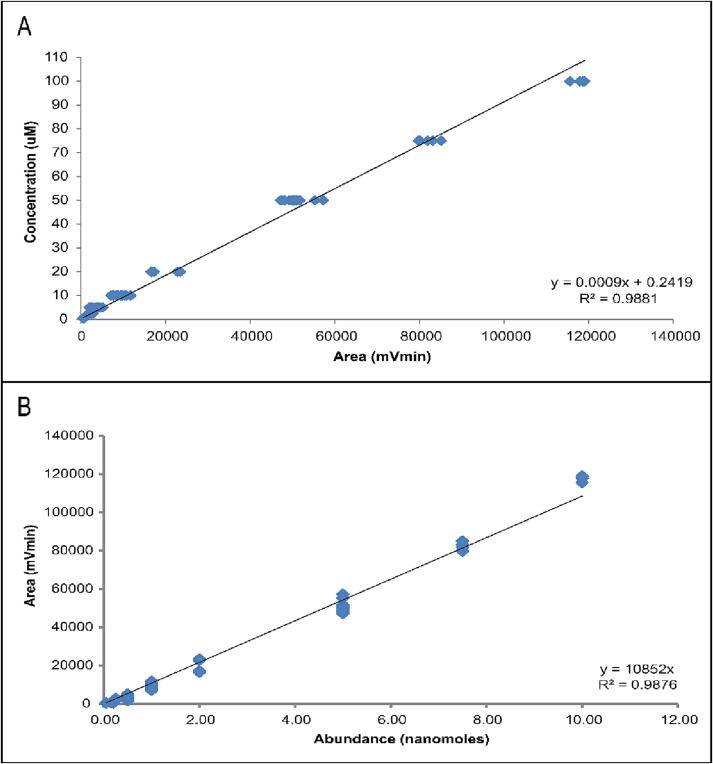
Calibration curves of the sodium nitrate standard, n = 89, T = 70–75 °C. A) The concentration for unknown samples may be determined using the linear equation of the trendline. The intercept of 0.2419 µM is the background NO_x_ concentration (15 PPB). B) The injected NO_x_ abundance against the measured mVmin response.

To demonstrate the efficacy of GNOME, we conducted multiple calibration curves on multiple analytical days at multiple temperatures. Following the Operation Instructions in Section 6 above, we present the analytical data and resulting calibration curves using an in-house sodium nitrate standard in Table 3 and Fig. 15 respectively.

**Table 3:** Analytical results of the sodium nitrate standard. The sample throughput of GNOME was determined using the time between injections obtained from the 20 same volume with same concentration (i.e., Day 3, C = 50 µM, V = 100 µL) injections.

Day	T (^o^C)	Standard concentration (µM)	Injected volume (µL)	Area (mVmin)	Abundance (nmoles)	Time between injections (mm:ss:ms)
1	75	0.5	300	743	0.15	–
1	75	0.5	300	696	0.15	–
1	75	5	100	5008	0.50	–
1	75	5	100	5166	0.50	–
1	75	5	100	4227	0.50	–
1	75	5	100	3845	0.50	–
1	75	10	100	11,816	1.0	–
1	75	10	100	11,729	1.0	–
1	75	10	100	8686	1.0	–
1	75	10	100	8735	1.0	–
1	75	20	100	22,798	2.0	–
1	75	20	100	23,475	2.0	–
1	75	50	100	57,258	5.0	–
1	75	50	100	55,326	5.0	–
2	75	0.5	100	501	0.050	–
2	75	0.5	100	536	0.050	–
2	75	0.5	300	688	0.15	–
2	75	0.5	300	654	0.15	–
2	75	2.5	100	2845	0.25	–
2	75	2.5	100	2844	0.25	–
2	75	2.5	200	2833	0.50	–
2	75	2.5	200	2888	0.50	–
2	75	5	100	4296	0.50	–
2	75	5	100	4516	0.50	–
2	75	5	100	4300	0.50	–
2	75	5	100	4313	0.50	–
2	75	10	100	10,425	1.0	–
2	75	10	100	10,811	1.0	–
2	75	10	100	9824	1.0	–
2	75	10	100	9486	1.0	–
3	70	50	100	50,333	5.0	01:50.0
3	70	50	100	50,081	5.0	01:39.8
3	70	50	100	50,495	5.0	01:45.8
3	70	50	100	51,585	5.0	01:46.5
3	70	50	100	50,890	5.0	01:45.0
3	70	50	100	50,962	5.0	01:48.4
3	70	50	100	50,561	5.0	01:49.0
3	70	50	100	50,534	5.0	01:39.8
3	70	50	100	50,954	5.0	01:48.1
3	70	50	100	49,229	5.0	01:47.4
3	70	50	100	47,482	5.0	01:44.7
3	70	50	100	50,163	5.0	01:50.6
3	70	50	100	50,151	5.0	01:41.2
3	70	50	100	50,990	5.0	01:50.2
3	70	50	100	51,718	5.0	01:46.6
3	70	50	100	48,188	5.0	01:38.8
3	70	50	100	49,859	5.0	01:45.5
3	70	50	100	50,078	5.0	01:48.2
3	70	50	100	50,869	5.0	01:49.9
3	70	50	100	47,194	5.0	01:42.2
3	70	75	100	83,153	7.5	–
3	70	75	100	80,128	7.5	–
3	70	75	100	79,784	7.5	–
3	70	75	100	81,898	7.5	–
3	70	75	100	85,160	7.5	–
3	70	100	100	115,607	10	–
3	70	100	100	117,972	10	–
3	70	100	100	119,054	10	–
3	70	100	100	118,654	10	–
3	70	100	100	117,926	10	–
4	75	0.5	400	543	0.20	–
4	75	0.5	400	626	0.20	–
4	75	2.5	200	2372	0.50	–
4	75	2.5	200	2104	0.50	–
4	75	5	100	2666	0.50	–
4	75	5	100	2014	0.50	–
4	75	5	100	3489	0.50	–
4	75	5	100	3916	0.50	–
4	75	10	100	8648	1.0	–
4	75	10	100	8731	1.0	–
4	75	10	100	7659	1.0	–
4	75	10	100	7097	1.0	–
4	75	20	100	16,579	2.0	–
4	75	20	100	17,269	2.0	–
5	75	2.5	200	1731	0.50	–
5	75	2.5	200	1911	0.50	–
5	75	2.5	100	2424	0.25	–
5	75	2.5	100	2294	0.25	–
5	75	5	100	3824	0.50	–
5	75	5	100	2347	0.50	–
5	75	5	100	3648	0.50	–
5	75	5	100	2732	0.50	–
5	75	10	100	8373	1.0	–
5	75	10	100	7614	1.0	–
5	75	10	100	8373	1.0	–
5	75	10	100	7614	1.0	–
5	75	20	100	16,715	2.0	–
5	75	20	100	16,723	2.0	–
5	75	20	100	16,715	2.0	–

### 7.1 Sample throughput and washout time

Using a suite of 20 same-sample and volume injections (100 µL of 50 µM sodium nitrate, 5 nmol abundance), the throughput rate is ∼34 samples/hour. This throughput corresponds to a response time of ∼15–17 s between samples (n = 20). The response time is defined as the time from injection to chemiluminescent detection and integration readout. Compared to the same-volume sample throughput in [8], the same-volume sample throughput of GNOME is 184 % larger. It should be mentioned that this sample throughput could be related to the chemiluminescent analyzer unit. In whichever case, GNOME is not the rate-limiting step regarding sample throughput. On the chemiluminescent analyzer used herein, we were able to successfully inject a new sample once the analyzer was detecting under 0.48 µM total NO_x_ without any sample peak overlap.

Given the predictability of the system washout, the GNOME could be further expanded by including an autosampler (or peristaltic pump if pump speed (mL/sec) is known) upstream of the injection port. This approach could improve sample throughput, particularly for consistent sample volume injections.

### 7.2 Dynamic range and calibration curve

The analytical data provided in Table 3 are shown as the calibration curves in Fig. 15. A total of 89 datapoints are compiled to construct the calibration curve. All data was collected over 5 separate analytical days (Table 3), indicating that the operation of GNOME was reproducible. Taking the mVmin area of the 20 same-sample and volume injections as an example, the average area of these samples is 50,116 mVmin ± 2458 mV min at the 2 σ level. This corresponds to a relative uncertainty of 5 % over the elapsed 35-minute analytical session. It is possible that very large volume samples, or very small volume samples would result in a reduced throughput due to slow washout or slow return to baseline, respectively. The source of this uncertainty may be the result of complicating matrix effects, or wet gas. The concentration calibration curve (Fig. 15A) covers a domain of 0.5 μM to 100 μM, with a corresponding correlation coefficient (r^2^) of 0.9881 (r = 0.9940). Alternatively, Fig. 15B displays the calibration curve comparing the injected abundance (nanomoles) versus response (mVmin), and has an r^2^ of 0.9876 (r = 0.9938). The dynamic range of GNOME is linear (r^2^ ≈ 0.99) from 0.05 to at least 10 nmoles, using volumes much less than the ‘few ml’ as in [15]. Evaluating linearity using abundance instead of concentration is preferable as abundance is absolute and irrespective of volume units. For example, the lower limit of 0.05 nmoles may be reached if a volume of 1 L with a concentration 50 pM is injected. The volume needed to meet the 0.05 nmol NO_x_ abundance required by the chemiluminescent analyzer is untenable at the concentration.

## 7.3 Concluding remarks and limitations

GNOME is sensitive down to at least 0.05 nmoles, and provides a linear response to a minimum of 10 nmoles, with a typical background NO_x_ concentration of 0.2419 µM. The upper limit is unknown, and is likely dependent upon the chemiluminescent analyzer used. The concentration range of the analyzer used for this study has a lower limit of ∼3 nM and an upper limit of ∼0.4 mM [14]. In conclusion the analytical capabilities of GNOME are:

• a resolution of 0.05 nmoles NO_x_ or lower are possible;
• a linear relationship up to a minimum NO_x_ abundance of 10 nmoles; and
• a throughput in excess of 30 samples/hr is possible for samples of moderate NO_x_ concentration (∼50 µM).

Suggested components which could be added to further improve reproducibility and sensitivity include (the following two suggestions are derived from [12]:

• a sodium carbonate column (could be packed in a glass tube with glass wool above and below the Na_2_CO_3_ with compression fittings. To avoid cracking the glass tube and losing the sample gas, plastic ferrules are suggested. This component would be located immediately downstream of the NaOH trap and serves to neutralize residual acidity; and
• a gas dryer (e.g., Perma Pure Nafion) located downstream of the Na_2_CO_3_ trap. The gas dryer requires a separate drying gas which flows against the direction of the sample gas. The counter flow drying gas is contained in a coaxial tube that surrounds the Nafion. Its purpose is to sweep away the H_2_O that is removed from the Nafion tubing.

A faster washout and increased sample throughput is possible if the tubing lengths of the gas handling line segments are reduced. Additionally, if the 700 mL condenser reservoir aa1 were replaced with a larger volume reservoir the condenser collection bottle could be better submerged in cold water-possibly improving the condensing capability. Moreover, if a larger condenser reservoir were used, the feed tube could be directed downwards-reducing splashing. Finally, larger sample volumes (i.e., >1 mL) should improve the NO_x_ area reproducibility to levels which have been reported elsewhere (e.g. [13]), although based on empirical observations, larger volume sample injections tend to produce chromatograms which are difficult to resolve due to slow washout as well as lingering elevated background NO_x_ levels.

## CRediT authorship contribution statement

**Samuel Bowman:** Conceptualization, Methodology, Data curation, Writing - original draft, Writing - review & editing.

## Declaration of Competing Interest

The authors declare that they have no known competing financial interests or personal relationships that could have appeared to influence the work reported in this paper.

## Appendix A Supplementary data

Supplementary data 1 xxx
